# Inflammation and Physical Performance in Overweight and Obese Schoolchildren

**DOI:** 10.3390/life14121583

**Published:** 2024-12-01

**Authors:** Cristina Popescu, Daniela Matei, Anca Maria Amzolini, Magdalena Rodica Trăistaru

**Affiliations:** 1 Doctoral School, University of Medicine and Pharmacy of Craiova, 200349 Craiova, Romania; popescu.crist@yahoo.com; 2 Department of Medical Rehabilitation, University of Medicine and Pharmacy of Craiova, 200349 Craiova, Romania; rodica.traistaru@umfcv.ro; 3 Department of Medical Semiology, University of Medicine and Pharmacy Craiova, 200349 Craiova, Romania

**Keywords:** children obesity, kinesitherapy program, physical performance, pro-inflammatory cytokines

## Abstract

Obesity represents a critical public health concern, often linked to low-grade chronic inflammation starting in childhood. This study aimed to evaluate the impact of a 12-week kinesiotherapy program on physical performance and levels of leptin and C-reactive protein (CRP) in overweight and obese children. Methods: Conducted at the Filantropia Municipal Clinical Hospital, 160 children aged 6 to 15 were randomly assigned to a study group (SG, n = 80) and a control group (CG, n = 80). The SG engaged in a tailored kinesiotherapy program, while the CG maintained their usual activities. All children and their families learned five key principles for preventing and managing obesity. Results: Results indicated significant improvements in the SG, with enhancements in the 6 min walking distance, Timed Up and Go test, and walking cadence (all *p*-values < 0.001). Notably, leptin and CRP levels (23.02 ± 7.17 to 16.62 ± 7.83, 4.13 ± 1.00 to 3.90 ± 0.95 mg/l, respectively) decreased significantly in the SG, contrasting with no significant changes in the CG. Regression analysis revealed a positive correlation between leptin levels and physical activity in the SG (coefficient: 0.5642, R-squared: 0.266). Conclusions: These findings suggest that targeted exercise programs can effectively enhance physical performance and reduce inflammation markers in overweight children, highlighting the importance of tailored interventions in managing obesity.

## 1. Introduction

Obesity is a complex condition caused by a combination of biological, behavioral, and environmental factors [1]. Many children today are growing up in environments that promote obesity, with easy access to high-calorie foods and a lack of physical activity, along with increased sedentary behavior, such as screen time [2].

Currently, overweight and obesity in children is a serious public health issue that the World Health Organization (WHO) has identified and widely discussed [3]. Childhood obesity is considered one of the most significant health challenges of the 21st century [4]. According to WHO, over 15% of children are overweight or obese [5]. The number of obese children increased rapidly until 2000, after which it plateaued, especially in developed countries. By 2025, it is estimated that 206 million children and adolescents (ages 5–19) will be living with obesity [6]. By 2030, it is predicted that nearly 30% of all children will be overweight or obese [7], leading to the conclusion that childhood obesity has reached pandemic proportions [8].

The American Academy of Pediatrics has identified two types of childhood obesity: metabolically healthy obesity (MHO) and metabolically unhealthy obesity (MUO) [9]. Children with MHO, despite being overweight, do not exhibit risk factors such as insulin resistance, high cholesterol, or high blood pressure [10,11].

Adipose tissue, often thought of as just an energy storage site, is actually a metabolically active organ that produces and releases biologically active compounds like inflammatory cytokines and acute phase reactants. In overweight children, excess fat leads to an imbalance of inflammatory molecules, increasing pro-inflammatory and decreasing anti-inflammatory ones, which contributes to the development of meta-inflammation [12,13]. New evidence shows that excess fat is linked to immune system changes, leading to chronic, low-grade inflammation [14]. This type of inflammation in obese children is similar to what is seen in obese adults [15]. The rise in obesity across all ages suggests that these inflammatory changes may begin early in life, shaped by genetics and early-life health factors [16]. Adipocytes produce important regulatory substances such as leptin, cytokines, components of the alternative complement pathway, and non-esterified fatty acids. These mediators allow adipocytes to communicate with systems like the pituitary–adrenal axis, sympathetic nervous system, and pituitary–gonadal axis [17]. Pro-inflammatory processes in adipocytes are essential for adipogenesis when consuming a high-fat diet [18] and reducing adipose tissue helps lower inflammation [19]. Fat cells produce key hormones and chemicals like leptin and cytokines, which regulate energy balance, metabolism, and the immune system [20]. Leptin, in particular, is involved in inflammation, while adiponectin has anti-inflammatory effects. Leptin levels increase with higher body mass index (BMI) [21,22], while adiponectin levels decrease. Leptin levels are also influenced by factors such as sex, age, and physical activity.

Since the 1970s, it has been recognized that fat tissue plays a role in linking inflammation with metabolism [23]. Fat tissue, which consists of fat cells, connective tissue, immune cells, and blood vessels, produces substances that are involved in both inflammation and metabolism [24]. Recent theories suggest that resistance to leptin, caused by excess fat, is the first step toward metabolic changes in obesity, leading to insulin resistance [25]. Although direct causal pathways are still under investigation, the evidence indicates that inflammation during early life predisposes individuals to metabolic [26] diseases later in adulthood.

Research on how kinesiotherapy affects leptin levels has shown mixed results in adults. As a non-pharmacological intervention, kinesiotherapy helps delay obesity-related comorbidities and reduces inflammation by lowering pro-inflammatory cytokines across all ages [27]. During kinesiotherapy, striated muscles adapt to exertion by increasing their mitochondrial count. This adaptation enhances the muscles’ ability to oxidize fatty acids, reducing their reliance on glycogen and blood glucose and lowering lactate production [28,29]. Some studies suggest that exercise lowers leptin levels, even without weight loss, while others found no effect. These conflicting results may be because the role of muscle as an endocrine organ, which also produces bioactive molecules during exercise, was not considered [30,31,32,33,34,35]. By decreasing visceral fat and reducing the release of adipokines, kinesiotherapy shows potential for being recognized as a long-term, effective treatment method [36]. Recently, Pranoto et al. [37] carried out a well-designed experimental study to assess how different types of kinesiotherapy affect pro-inflammatory cytokines. Researchers found that participants in moderate-intensity endurance training, moderate-intensity resistance training, and moderate-intensity combined training all experienced significant reductions in serum IL-6 and TNF-α levels after the training sessions. Notably, the study concluded that combined training is more effective at lowering pro-inflammatory cytokines compared to aerobic or resistance training conducted separately.

The WHO recommends that children between the ages of 5 and 17 engage in at least 60 min of moderate to vigorous physical activity daily [38]. This helps prevent obesity by increasing energy expenditure and improving cardiovascular health. The positive effects of exercise are also linked to its anti-inflammatory properties [38], and studies show that physical activity can significantly reduce leptin levels in children [39,40], regardless of exercise intensity [41].

Research consistently indicates that obese children display altered gait patterns, including asymmetries, which may contribute to musculoskeletal issues and reduced mobility [42]. It was shown that the kinetic asymmetries present might be a key predictor in knee osteoarthritis progression [43].

However, most of the studies tend to examine either inflammatory markers or gait parameters individually, without investigating how these two factors interact. The relationship between systemic inflammation and gait issues in obese children is still not well understood. Additionally, there is little research on how aspects like age or living environment might influence kinesiotherapy’s impact on cytokine levels and gait improvements in children. Gaining insight into these connections could help clarify how systemic inflammation affects movement, supporting the development of more comprehensive and targeted rehabilitation programs.

Recognizing that both fat and muscle tissue help regulate inflammation by lowering pro-inflammatory molecules and boosting anti-inflammatory ones [44], a randomized experimental trial was conducted. This pilot study aimed to evaluate the effects of a structured kinesiotherapy program on both inflammatory markers and key gait parameters in overweight and obese children. The study had a dual primary objective:

1. To determine the impact of a combined aerobic and walking coordination kinesiotherapy program on two inflammatory markers—leptin and C-reactive protein (CRP)—to explore potential benefits for inflammation management in obese children.
2. To assess the program’s effectiveness in improving key physical performance parameters, specifically the six-minute walk distance (6MWD), walking cadence, Timed Up and Go (TUG) test performance, and gait symmetry index.

The study introduces an innovative approach by incorporating walking coordination exercises into the kinesiotherapy program, which specifically target cadence and symmetry index—two gait parameters rarely emphasized in existing interventions for overweight and obese children. This tailored approach seeks to improve gait mechanics while addressing broader health outcomes such as inflammation and BMI changes.

Additionally, the study investigates subgroup variations based on age, gender, and living environment, offering a comprehensive perspective on how different demographic factors may influence inflammatory responses and physical performance outcomes. By addressing these interconnected elements, the research aimed to provide actionable insights for targeted interventions in pediatric obesity management.

## 2. Materials and Methods

### 2.1. Design Overview 

Our prospective, random study was conducted at the Pediatrics and Physical Medicine and Rehabilitation Departments of Filantropia Municipal Clinical Hospital in Craiova, from January 2022 to June 2024. The study involved 160 overweight and obese children admitted to the Pediatric Clinic who were assessed by a multidisciplinary team (T1). The 160 children in the study were randomly assigned to two groups (study and control). Block randomization was employed based on age, environment, and gender. Patients were assigned to groups according to the sequence within each block as they joined the study. To ensure accuracy, SPSS statistical software (version 20, IBM Corporation, Armonk, NY, USA) was used to help maintain balanced group sizes throughout the study. Blinding was not applied in this study due to the specific nature of the intervention, which allowed families to be aware of the treatment, facilitating adherence and monitoring of results. Steps were taken to minimize potential bias by using standardized evaluation protocols, including objective lab measurements, standardized functional assessment tests, and a tool for measuring gait parameters. Additionally, groups were analyzed by age, gender, and background to ensure consistency and internal validity of the results.

Results were reported as means, standard deviations (SDs), and *p*-values between the two groups. The effect size was calculated, and a power analysis was conducted. The results from the power analysis indicate that the study was sufficiently powered to detect meaningful differences in 6MWD, walking cadence, TUG, symmetry index, and CRP.

The patients were randomly divided into two groups: the SG (n = 80) and the CG (n = 80). To assess the lab parameters and short-term effects of the applied kinesiotherapy program, a follow-up evaluation was conducted 3 months from the beginning of the treatment (T2).

**Figure 1:**
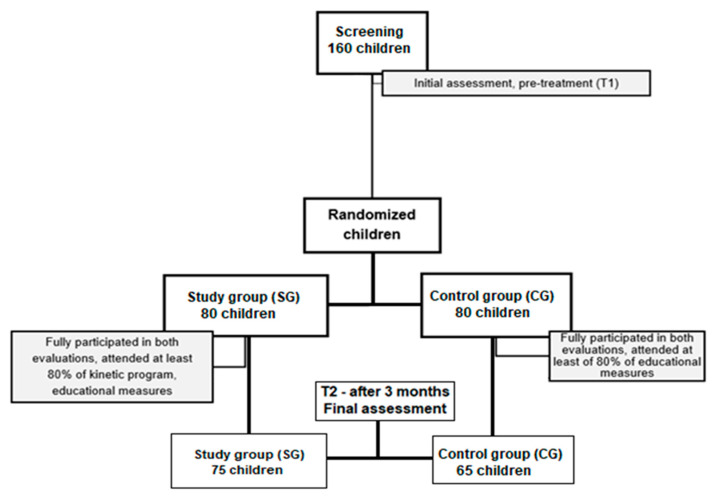
Diagram of the study design.

The children in the SG underwent a 12-week program designed to improve and maintain their functional status. This program included educational measures tailored to overweight and obese children, along with an age-appropriate exercise program based on each child’s compliance level. In contrast, the children in the CG continued their usual daily activities as before hospitalization but were advised to follow educational recommendations, such as maintaining a balanced diet, reducing screen time, and ensuring a proper sleep schedule. For the statistical analysis, only those who fully completed both evaluations, attended at least 80% of the kinesiotherapy, and provided valid data were included (Figure 1). In the SG, only 5 children (6.2%) did not complete the prescribed 12-week kinetic program or attend the second evaluation, whereas in the CG, 15 children (18.8%) did not attend the second evaluation.

### 2.2. Participants

The inclusion criteria were as follows: (1) children aged 6 to 15 years; (2) written consent from a parent or guardian, along with verbal assent from the child; (3) a BMI above the normal range; (4) a confirmed diagnosis of MHO; (5) the ability to speak and understand Romanian; (6) no previous experience with kinesiotherapy. The exclusion criteria included (1) any psychiatric disorders; (2) recent traumatic injuries; or (3) other conditions (orthopedic, respiratory, or neurological) that could hinder kinetic ability. Following these established criteria, eligible patients and their parents or guardians were informed about the study and given the appropriate informed consent forms.

In an attempt to deepen the proposed topic and maintain the kinesiotherapy approach, the patients were evaluated in various subgroups based on gender (girls/boys), the environment they came from (rural/urban), and age category (younger schoolchildren = 6–10 years, older schoolchildren = 11–15 years).

### 2.3. Study Treatment

The kinesiotherapy was initiated at the Physical Medicine and Rehabilitation Department of Filantropia Hospital in Craiova, under the guidance of a specialized physiotherapist. Initially, each child was tested for their maximum aerobic training level. The kinetic program consisted of 60 min sessions of aerobic, flexibility, and gait coordination exercises, held three times a week for three months, in addition to the regular physical education classes each child attended at school. The kinesiotherapy was chosen based on the child’s preferences and family availability, with intensity tailored by the physiotherapist according to the child’s physical activity level, following guidelines from Ainsworth et al.’s “Physical Activity Compendium” [45]. The intensity of aerobic training exercises was managed by adjusting the frequency and duration of the physical movements involved in each exercise. The submaximal level of aerobic training was represented by a value ranging between 75 and 80 percent of the predetermined maximum intensity for each child during the initial assessment.

Every two weeks, progress was monitored by the same physiotherapist to ensure proper execution of the exercises, particularly focusing on gait coordination and flexibility, including stretching. To keep the aerobic programs consistent, the intensity was controlled. Each 10 min segment of exercise included at least 5 min of moderate-to-high intensity activity and 2 min of vigorous intensity activity (Table 1). The exercises were designed as games to make them more enjoyable for the children, with variations in the specific exercises used. Outdoor activities were preferred, but if the weather was bad, they were held indoors. Fundamental movement skills like running, skipping rope, and swimming were gradually introduced, progressing at a controlled, individualized pace, as recommended in the literature [46]. Additionally, the physiotherapists encouraged the children to ride bicycles, walk to and from school, and play in the nearby park. These seemingly small activities are vital to promoting a healthy lifestyle in children [47,48].

All the children in this study and their families were informed about five key principles for preventing and managing childhood obesity, based on Centers for Disease Control and Prevention guidelines [49]. These include “Eat the Rainbow” (a diet rich in fruits, vegetables, and fiber), “Move More” (regular physical activity), “Slow Down on Sugar” (limit sugar intake), “Reduce Screen Time” (limit time on screens like mobile devices and TV), and “Sleep Well” (9–12 h of sleep for children 6–12 years old and 8–10 h for teens 13–18). The pediatric doctor continuously monitored these educational aspects, documenting them in each child’s file.

By determining the maximal effort level for each subject and performing kinesiotherapy exercises at a submaximal level, the goal was to avoid potential causes of therapy dropout, such as physical discomfort or fatigue. Additionally, varying exercises within the same category of kinesiotherapy aimed to prevent loss of interest or motivation. Involving families in understanding general guidelines for supporting children with obesity was intended to reduce psychological or social factors that might affect adherence to the kinesiotherapy program.

**Table 1:** Adapted physical exercise program (kinesiotherapy)(SG).

Kinetic Objective	Description	
10 min warm-up—free walking, with balance of the upper limbs		
Flexibility exercises	Waltz steps or another agreed dance	Daily, 10 min
	Gymnastic exercises/jumping rope, rolling	Daily, 10 min, 5 sets of 2 min
Aerobic exercises (restoring/maintaining the ability to exercise, beneficial impact on the bone structure)	Child-friendly sport	60 min/session
	(basketball, volleyball, tennis, swimming, running)	3 sessions/week
	House cleaning	60 min/activity
	Bike ride for non-sports people	3 sessions/week
Walking coordination exercises	Walking in tandem	Daily, 15–20 min
	Biofeedback in the mirror Walking the pet	
10 min cool-down—easy running, stretching of the muscle groups of the lower limb		
Strength exercises were not indicated; the ages of the children were under 16 years. Strength exercises are recommended in adolescents after 16 years [50].		

Based on general recommendations, we used several types of exercises adapted to each child, as follows:

1. Jumping rope:
  It was conducted daily, with the request for jumps to be as wide as possible after the child had mastered the technique. For each exercise, the child jumped in a certain way with their lower limbs.
  Examples:
  - Jumped on the right foot once.
  - Jumped alternately with each foot, once.
  - Jumped with crossed feet, three times.
  - Jumped twice on the left foot and twice on the right foot.
  - Jumped with both feet together, supported on both.
  - Jumped with crossed feet (hyper-adducted).
  - Jumped with feet abducted, landing on both soles.
  - Jumped alternately on each foot.
  - Jumped on both feet, but with the rope taken backward, in the opposite direction.
  - Free style, a combination of the previous variations.
2. Walking in tandem:

In the kinetic hall, a straight line (with a length of 6 m) was marked on the ground using colored tape. The child patient was instructed to stand at one end of the line. Then, the child was asked to place their right foot directly in front of their left foot, ensuring that the heel of the front foot touched the toes of the back foot. The child was encouraged to walk slowly and maintain their balance. To increase difficulty, the child was asked to walk backward or with their eyes closed.

After 2 weeks, obstacles or challenges were introduced, such as balancing a sandbag (1 kilo) on their head while walking.

The session time = 15 min/daily.

3. Biofeedback in the mirror:
  In the physical therapy room, exercises were conducted under the supervision of a physical therapist, using a large mirror placed in a well-lit area to allow children to observe their reflections clearly. During the activity, each child stood in front of the mirror and performed specific movements, such as the following:
  - Balancing on one foot.
  - Reaching for an object: this involved reaching to the side for a weight (250 g or 500 g) placed at variable heights, then moving the object to the opposite side.
  - Observing posture and alignment: after completing each movement, the child checked their posture and body alignment in the mirror.

The child was encouraged to describe their observations. For example, “Are your shoulders level? Is your back straight?” The therapist provided positive reinforcement and offered corrections based on these observations, such as “Try to keep your knee aligned with your toes”. Children were encouraged to try different positions and movements, using the mirror as a guide. This exercise not only developed physical skills but also enhanced each child’s self-awareness and confidence in their movements.

The session time = 15 min/daily.

### 2.4. Ethical Considerations

The primary considerations for this investigation were ensuring the patients’ safety and well-being. Eligible patients and their parents were briefed about the study and given the required informed consent forms. The parents or guardians of the children participating in the study signed the consent form after being fully informed about the study’s purpose, the benefits and drawbacks of participation, potential side effects, the confidentiality of personal data, and the patients’ right to withdraw from the study at any time without needing to provide a reason or face any consequences. The protocol was carried out in accordance with the Declaration of Helsinki and Good Clinical Practices and was approved by the Ethics Committee (opinion no. 38/1 March 2022).

### 2.5. Collecting Information Methods

The following parameters were considered: *anthropometric data* from the clinical exam, *lab results*, and *physical performance tests*.

Anthropometric data: height and weight were measured, and BMI was calculated using the formula BMI = weight (kg)/height^2^ (m^2^). Body weight was recorded using a Fazzini S7350HR electronic scale with a range of 0 to 200 kg and an accuracy of 100 g. Height was measured with a portable stadiometer with 0.1 mm accuracy. The children were barefoot and wore light clothing (shirt and shorts) during the measurements. Each measurement was taken three times by the same evaluator, and the average value was used. The study followed the International Obesity Task Force (IOTF) BMI cutoffs [51], which define underweight, overweight, and obesity based on BMI thresholds of 18.5, 25, and 30 kg/m^2^ at age 18.

The laboratory tests included a general blood test, CRP, lipid profile, and biochemical markers like leptin, using commercial kits from Biovendor R&D (Brno, Czech Republic) based on the sandwich enzyme-linked immunosorbent assay (ELISA) technique. Blood samples were collected from the antecubital veins of all patients after an overnight fast, following aseptic procedures. The tests were conducted using two automatic analyzers (MINDRAY BC-6800 for blood tests and ARCHITECT C4000 for biochemical analysis). Standard reference values were used to interpret the results, including neutrophils (1.5–8.0 × 103/μL), lymphocytes (3–8 × 103/μL), platelets (150–450 × 103/μL), monocytes (0.00–1.00 × 103/μL), CRP (0–0.5 mg/dL), total cholesterol (120–170 mg/dL), HDL cholesterol (50–130 mg/dL), triglycerides (35–150 mg/dL), blood sugar (60–100 mg/dL), and leptin levels (3.63–11.09 ng/mL for girls, 2.05–5.63 ng/mL for boys). Normal leptin values in the literature are 7.5–9.3 ng/mL for normal-weight children and 24.1–31.3 ng/mL for obese children [52].

Regarding the physical performance tests, for gait analysis, the following methods were used. The BTS G-WALK wireless system (BTS Bioengineering Corp., Garbagnate Milanese, Italy) was used, which includes an inertial sensor made up of a tri-axial accelerometer, magnetic sensor, and tri-axial gyroscope. This device, worn by the patient, allows for a functional analysis of gait. Each child was instructed on how to properly perform the physical performance tests and was familiarized with the equipment. Verbal encouragement was provided during the tests to promote optimal effort. Gait parameters were measured to assess the physical performance of overweight and obese children before and after the exercise program. These included the following:

- TUG test: The child starts seated in a chair, stands up, walks 3 m (marked by a line), crosses it, turns, and returns to the chair, without assistance. The time taken to complete the task is recorded. This test provides insight into gait stability, balance during transitions, and overall gait changes.
- Symmetry index: This measures the child’s ability to maintain a similar pattern of acceleration and deceleration of their center of mass during both sides of the gait cycle. A score close to 100 indicates good symmetry, with non-pathological subjects typically scoring over 90.
- 6MWT: This test evaluates the distance walked in six minutes (6 MWD) and walking cadence (steps/min). It was performed indoors [53] along a 20 m corridor, marked every 2 m. Each child was instructed to walk as far as possible in 6 min, with minimal verbal encouragement (“keep going” and “doing well”). This test is simple, cost-effective, and highly useful for assessing exercise capacity.
- Walking cadence: This measures the number of steps taken per minute. A faster cadence with shorter steps helps distribute forces evenly across the lower joints, creating a biomechanical balance, especially important for children with excess weight.

### 2.6. Statistical Analysis

The database was created and analyzed using SPSS software (version 20, IBM Corporation, Armonk, NY, USA). Quantitative data were summarized as means and standard deviations (SDs). To assess within-group changes over time for physical performance, serum leptin and CRP paired-sample Student’s *t*-tests were used. For comparisons between the SG and CG, the t-test for independent samples with unequal variances was applied.

The Wilcoxon signed-rank test was performed to check for significant differences between the mean and median of each parameter in both groups. All *p*-values were 1.0, indicating no significant difference between the mean and median, suggesting a symmetric data distribution.

Pearson correlations were calculated between initial and final values for all parameters, with significance set at *p* < 0.05 (*p* < 0.01 for highly significant differences). Finally, the ANOVA (Analysis of Variance) test was used to evaluate differences in physical performance between initial and final values for each group. A *p*-value less than 0.05 was considered statistically significant.

## 3. Results

### 3.1. The Demographic and Anthropometric Data

The children’s anthropometric data are shown in Table 2. The average age in both groups is similar, with 9.22 years for the SG and 9.89 years for the CG, a difference of just 0.67 years, which is not statistically significant (*p* = 0.1347). The urban-to-rural ratio is 0.92 for SG and 1.09 for CG, again without significant differences between groups. In the SG, there are 9 more boys than girls, while in the CG, the ratio of girls to boys is roughly equal. When comparing BMI by gender and area of residence, no significant differences were found (*p* = 0.7707 for girls and *p* = 0.4158 for boys; *p* = 0.2876 for urban children and *p* = 0.7612 for rural children), meaning that where the children live does not impact the condition studied. This was also true across schooling levels, except for high school students in the CG, who were classified as obese while other school categories were classified as overweight.

**Figure 2:**
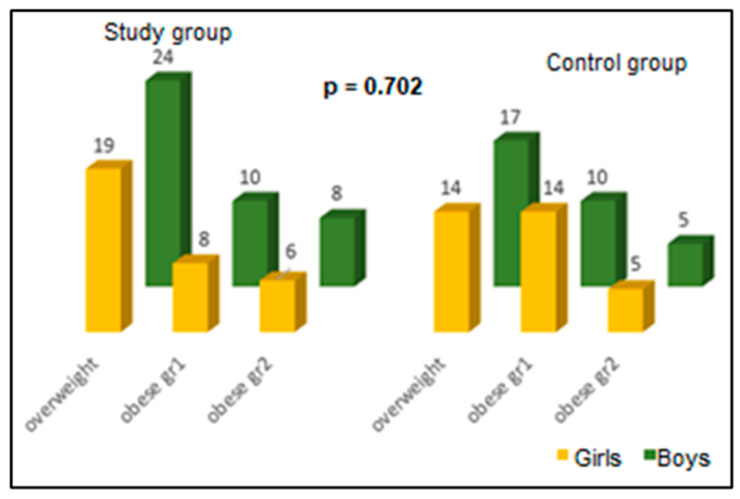
Classification of children by obesity and gender groups (*p* = the *t*-test for independent samples).

Depending on BMI, overweight children predominated (Figure 2). The numerical differences between girls and boys in the two groups are not significant (*p* = 0.702).

### 3.2. The Laboratory Tests

**Figure 3:**
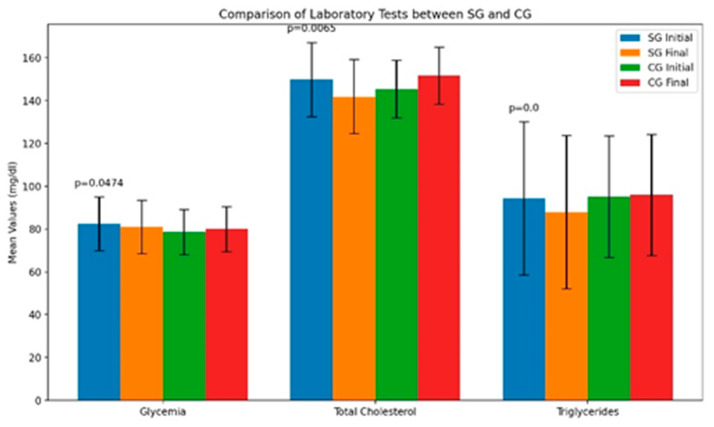
The laboratory tests in all children. Mean values and standard deviations (*p* = the *t*-test for independent samples).

Referring to the entire study group, the average lab test results were within normal ranges at both evaluation points (see Table 3 and Figure 3), supporting their classification as MHO. The average CRP levels (standard test) were below the normal limit of 5 mg/L in both groups. Only leptin levels were above normal, but they did not exceed 24.1 ng/mL, which is considered the lower limit for obese children (see Table 4).

Study group (SG): In this group, significant improvements were observed across several metabolic and inflammatory parameters after the kinesiotherapy intervention:

- Glycemia: There was a significant decrease in blood glucose levels from the initial assessment (T1) to the final assessment (T2) (*p* = 0.0474). This indicates improved blood sugar regulation among the participants following the exercise program.
- Total cholesterol: Participants showed a significant reduction in total cholesterol levels after the intervention (*p* = 0.0065), suggesting a positive impact on lipid metabolism and a potential reduction in cardiovascular risk.
- Triglycerides: There was a marked decrease in triglyceride levels (*p* < 0.001), further supporting improvements in metabolic health and the effectiveness of the exercise program in managing lipid profiles.
- Leptin levels: The levels of leptin, a hormone associated with appetite regulation and fat storage, demonstrated a significant reduction at T2 compared to T1 (*p* < 0.001). This reflects decreased inflammation and may indicate improved energy balance and reduced adiposity.
- C-Reactive protein (CRP) levels: Although CRP levels were within normal limits, they showed a significant decrease after the intervention (*p* < 0.001). This suggests a reduction in subclinical inflammation, which is often associated with obesity and can contribute to long-term health risks.

These findings highlight the positive impact of the kinesiotherapy program on the metabolic and inflammatory health of overweight and obese children in the study group. The significant improvements in glycemia, lipid profiles, and inflammatory markers underscore the effectiveness of structured physical activity interventions in managing childhood obesity and its associated health complications.

Control group (CG): In contrast, the control group, which did not participate in the kinesiotherapy program, showed minimal changes:

- Total cholesterol: This was the only parameter that exhibited a statistically significant change between T1 and T2 (*p* = 0.043). However, the change was less pronounced compared to the study group.
- Leptin and CRP levels: The levels of leptin and CRP remained virtually unchanged over the study period, suggesting that without the intervention, there was no significant impact on inflammation or metabolic health.

This lack of significant improvement in the control group emphasizes the role of the kinesiotherapy program in effecting the positive health changes observed in the study group.

Between-group comparisons: In the baseline assessment (T1), significant differences were noted between the study group and the control group in several parameters:

- Glycemia: The study group had significantly different blood glucose levels compared to the control group (*p* = 0.05).
- Total cholesterol: There was a significant difference in total cholesterol levels between the two groups (*p* = 0.009).
- Leptin levels: Significant differences were observed in leptin levels (*p* = 0.0164), indicating variations in inflammation and adiposity between the groups at the start of the study.
- CRP levels: Differences in CRP levels were also significant (*p* = 0.0261), suggesting differing levels of subclinical inflammation between the groups.

By the end of the study (T2), these differences became even more pronounced:

- Leptin levels: The study group showed significantly lower leptin levels compared to the control group (*p* = 0.009), indicating a greater reduction in inflammation and potentially improved metabolic function due to the intervention.
- CRP lLevels: Highly significant differences were observed in CRP levels (*p* < 0.001), with the study group demonstrating a substantial decrease, reinforcing the effective-ness of the kinesiotherapy program in reducing subclinical inflammation.

**Figure 4:**
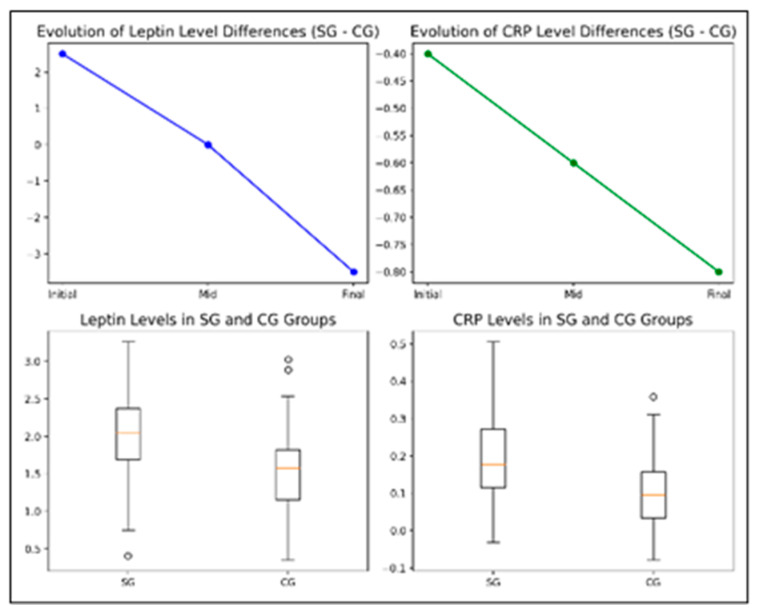
Comparative analysis of Leptin and CRP levels in SG and CG groups over time (paired-sample Student’s *t*-tests).

Figure 4 presents combined box plots and line graphs illustrating leptin and CRP levels in the SG and CG groups, highlighting significant differences in central tendency, variability, and temporal trends. At baseline, the SG group exhibited higher variability in leptin levels, with more outliers and a slightly lower median CRP level compared to the CG group. Over time, the SG group experienced significant reductions in both leptin and CRP levels following the kinesiotherapy program, indicating its positive impact on sub-clinical inflammatory status. Conversely, the CG group maintained relatively stable leptin and CRP levels throughout the study. These findings, supported by statistically significant results from *t*-test and ANOVA analyses, underscore the effectiveness of the intervention in improving inflammatory markers.

Table 4 summarizes the significant changes in leptin and CRP levels across various subgroups. In the SG, both leptin and CRP levels demonstrated significant reductions between the two evaluation points, with consistent trends observed across gender (leptin: *p* = 0.0000 for boys, *p* = 0.0001 for girls; CRP: *p* = 0.000 for both), environment (leptin and CRP: *p* = 0.000 for urban and rural), and age groups (leptin: *p* = 0.000 for younger, *p* = 0.01 for older; CRP: *p* = 0.000 for both).

In the CG, CRP levels also showed significant reductions regardless of gender (*p* = 0.000) or environment (*p* = 0.000), while leptin levels decreased significantly among boys (*p* = 0.004) and children from urban areas (*p* = 0.001).

Between-group comparisons at the T2 evaluation revealed significant differences in CRP levels across all subgroups: gender (boys: *p* = 0.0022, girls: *p* = 0.0001), age groups (younger and older: *p* = 0.000), and environments (urban: *p* = 0.005, rural: *p* = 0.00004). Similarly, leptin levels were significantly different, particularly among boys (*p* = 0.0019), younger children (*p* = 0.002), and participants from urban (*p* = 0.03) and rural (*p* = 0.0072) areas.

These findings highlight the broad effectiveness of the kinesiotherapy program in reducing inflammatory markers, with notable differences across gender, age, and environment.

**Figure 5:**
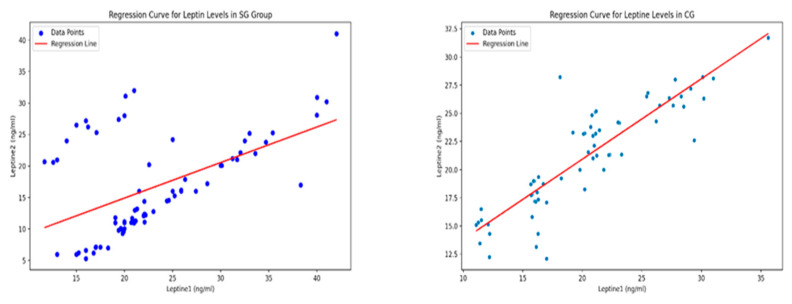
The regression plot for visually representing the relationship between leptin levels for each group of children, confirming the model’s findings (paired-sample Student’s *t*-tests).

The regression analysis (Figure 5) for the SG shows a moderately strong positive relationship between leptin levels at the T1 and T2 evaluations, with a coefficient of 0.5642. The R-squared value of 0.266 means that about 26.6% of the variability in Leptin T2 is explained by Leptin T1, suggesting that other factors, like physical activity, may also influence Leptin T2 levels. In the CG, the data show a stronger relationship between the two time points, with a slope of 0.71 and an R-squared value of 0.76, indicating that changes in Leptin T1 are closely linked to changes in Leptin T2, even though CG children did not participate in the supervised kinesiotherapy program.

### 3.3. Gait Analysis—Physical Performance Tests

#### 3.3.1. Study Group

**Figure 6:**
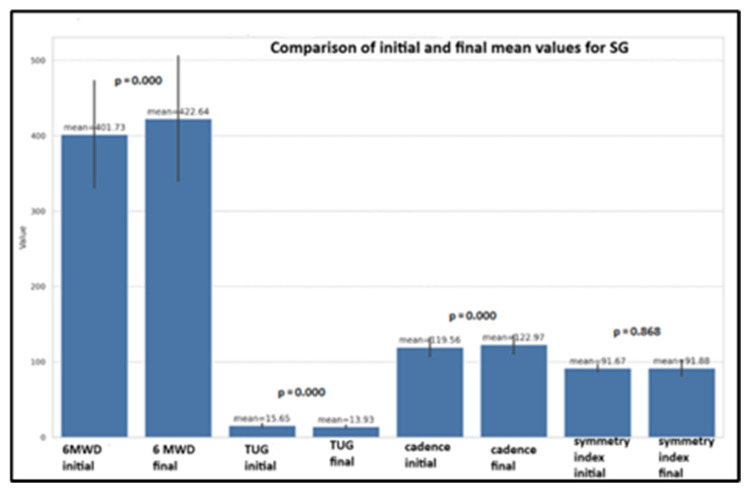
The physical performance tests for SG children (mean, SD, *p* for *t*-test).

The statistical analysis shows significant improvements in the 6MWD (*p* = 0.000), TUG (*p* = 0.000), and walking cadence (*p* = 0.000) for the SG group (T1/T2 evaluation), confirming that the kinesiotherapy was effective in boosting these specific physical performance measures. However, the symmetry index did not show any notable change (*p* = 0.868) (Figure 6).

The statistical analysis revealed significant improvements in physical performance measures for the SG group between the T1 and T2 evaluations. The 6MWD, TUG, and walking cadence all showed highly significant changes (*p* = 0.000), confirming the effectiveness of the kinesiotherapy program in enhancing these specific measures. In contrast, the symmetry index remained largely unchanged (*p* = 0.868), indicating its relative stability over the intervention period (Figure 6).

Subgroup analysis showed notable differences in physical performance measures across age groups, with the exception of walking cadence in rural children and the symmetry index in both older schoolchildren and rural children. Although the symmetry index decreased slightly, it remained above 90%, reflecting the intricate motor control processes in adolescents, which may require more targeted interventions to improve.

**Figure 7:**
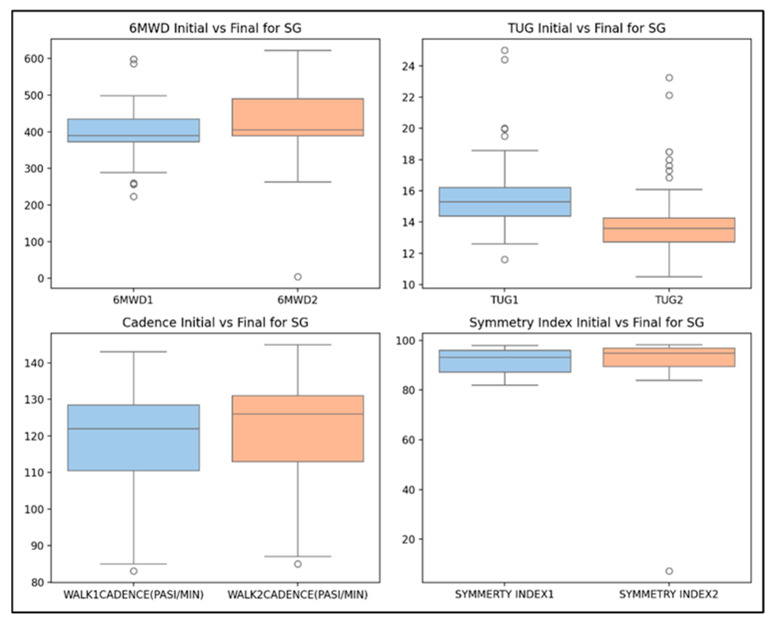
The box plots for physical performances in SG children (paired-sample Student’s *t*-tests).

The improvements across all four parameters, albeit minimal for the symmetry index, are further illustrated in Figure 7. The box plots highlight significant gains in the 6MWD, TUG, and walking cadence from baseline to final evaluations, reinforcing the positive impact of the kinesiotherapy program on physical performance in school-aged children.

**Figure 8:**
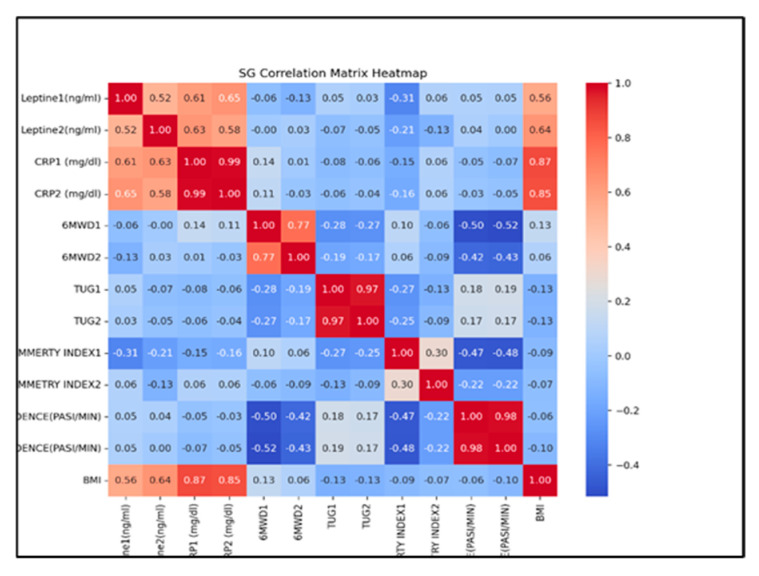
The correlation matrix for SG (Pearson correlations).

The SG group correlation matrix (Figure 8) reveals strong positive correlations between related variables, such as leptin levels, CRP levels, 6 min walk distance, Timed Up and Go, and walk cadence, indicating consistent measurements across these variables. Additionally, the positive correlation between BMI and CRP, respectively, and leptin levels suggest a link between obesity and inflammation.

#### 3.3.2. Control Group

**Figure 9:**
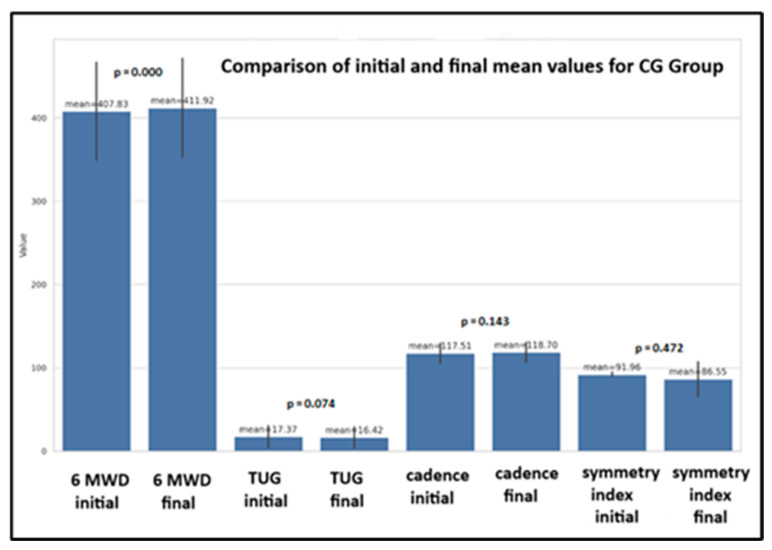
The physical performance tests for CG children (mean, SD, *p* for *t*-test).

For CG, the statistical analysis (Figure 9) shows significant improvements only in the 6MWD (*p* = 0.000) for all children, regardless of age group, gender, and environment. Also, the significant differences were seen only in two parameters: TUG for girls (*p* = 0.000) and walking cadence (*p* = 0.000), both in girls and rural areas.

**Figure 10:**
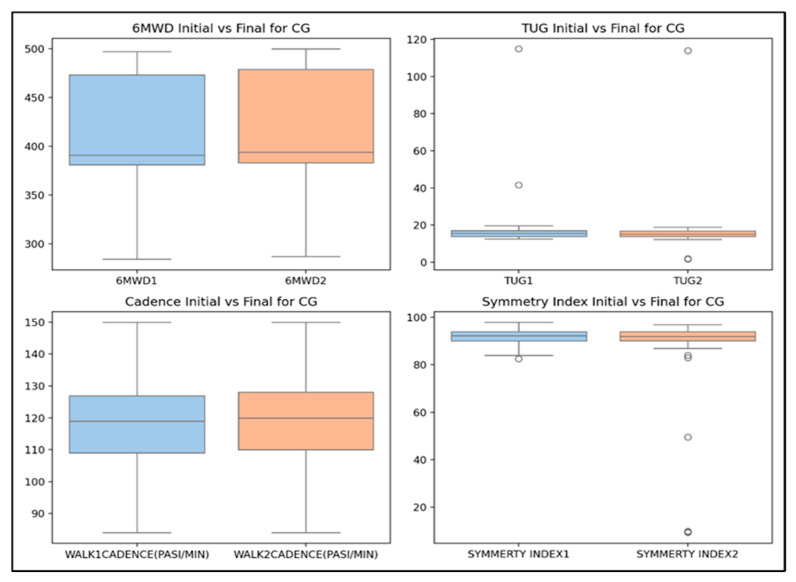
The box plots for physical performances in CG children (paired-sample Student’s *t*-tests).

The median values for both the initial and final measurements for CG children (Figure 10) are relatively close; the spread of the data remains consistent for all parameters, indicating minimal change and suggesting stable performance among participants. These results are explained by the absence of the kinesiotherapy program.

#### 3.3.3. Intergroup Analysis

At T1, no statistically significant differences were observed between the studied parameters in the two groups. By T2, significant differences emerged for walking cadence (Table 5) and symmetry index (Table 6).

Specifically, in older children, 6MWD showed a significant improvement (*p* = 0.000), while walking cadence improved significantly in both older (*p* = 0.032) and younger children (*p* = 0.005). Gender and environment comparisons revealed notable differences only in the initial symmetry index for boys (*p* = 0.0471) and walking cadence for girls (*p* = 0.018). Overall, no other significant differences in physical performance were observed between the groups (*p* ≥ 0.05).

**Figure 11:**
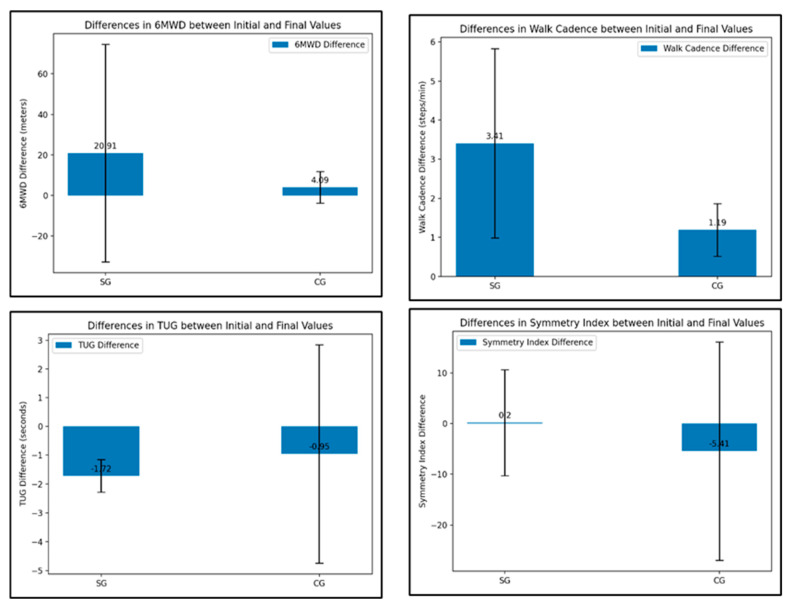
The ANOVA test results for the four gait analysis—physical performance parameters differences between the SG and CG groups.

The *ANOVA test* on the differences between initial and final performance parameters values for each group was performed. There is a statistically significant difference in the changes of 6MWD (*p* = 0.013), walking cadence (*p* = 0.000), and symmetry index (*p* = 0.047) over time within each group. For TUG, differences between the SG and CG groups show a *p*-value of 0.085, indicating no statistical significance (Figure 11).

## 4. Discussion

In this research, the physical performance of overweight and obese children who followed a customized kinesiotherapy program was assessed and tracked. We also examined how leptin, a key marker of inflammation in childhood obesity, was affected. While the effects of physical exercise on inflammatory factors in obese children can be uncertain [54], it is well established that exercise reduces cardiovascular risk and insulin resistance. Thus, the levels of CRP and leptin before and after the exercise program were measured.

Research [55,56] suggests that certain dietary patterns can impact pro-inflammatory markers like CRP in obese children. Studies indicate that a diet rich in fruits, vegetables, and whole grains and low in sugary drinks and processed meats may help lower CRP levels and reduce inflammation. These findings are relevant to preventing and managing obesity-related complications in children. In terms of our study’s results, since the investigators only provided dietary recommendations without actively monitoring the participants’ diets, the authors cannot draw firm conclusions about the diet’s impact on the levels of the measured markers we measured. Previous research has shown that regardless of the duration of the kinesiotherapy program—whether two [57], three [58], or eight months [59]—some studies found no changes in CRP and other inflammatory markers (like fibrinogen, IL-6, and serum adiponectin), despite improvements in BMI and fitness levels. However, Balagopal et al. [60] reported a decrease in CRP and IL-6 after just three months of kinesiotherapy, though their study had a small sample size. Significant differences in average CRP values for the study group were observed, indicating that the kinesiotherapy program positively affected subclinical inflammation related to excess weight. The research studies [61] suggest that kinetic programs, such as exercise and physical activity, can have a positive impact on leptin levels, physical performance, and overall health in obese children. Regular physical activity and exercise training can improve physical performance, reduce leptin levels, and enhance body composition in obese children. These findings have implications for the development of effective interventions to improve physical performance and reduce the risk of obesity-related complications in children. Although the serum leptin levels were elevated, our participants did not have metabolic syndrome, with average values being above normal but lower than those typically found in obese individuals. This can be attributed to the fact that our study focused mostly on overweight children. Our findings support the idea that leptin contributes to subclinical inflammation in obese children [62].

The lack of significant correlations between leptin levels and average blood glucose and lipid parameters can be explained by the specific characteristics of our participants. However, similar results have been observed in children with metabolic syndrome [63]. Since 2006, a positive correlation between serum leptin levels and adipose tissue mass has been established, highlighting a linear relationship between BMI and basal leptin levels. In our study, the average serum leptin levels significantly correlated with BMI. Therefore, our findings concerning CRP and leptin levels reinforce the idea that obesity-related inflammation begins early in childhood [64].

Leptin, a key adipokine primarily produced by fat cells, helps regulate energy balance [65]. Kinesiotherapy can positively influence this balance. Our study demonstrates that a well-structured exercise program effectively improves the physical performance of overweight and obese children and leads to favorable changes in their leptin levels.

The authors emphasized participation in organized sports, as they have proven to be more effective in reducing weight and complications from pediatric obesity than individual exercises [66]. The duration, intensity, and type of exercise significantly affect the redistribution of immune cells in the bloodstream [67]. Our program lasted 12 weeks, based on literature studies suggesting that regular training over 8 weeks can reduce pro-inflammatory cytokines and lower systemic inflammation, along with improving insulin sensitivity.

The intensity of the exercises in our program was submaximal because the children in the SG could not consistently perform high-intensity exercises. Therefore, the results based on this submaximal intensity were evaluated. We believe that the leptin levels changed significantly, consistent with other studies. For instance, Tenòrio et al. [44] found that both low- and high-intensity exercises could significantly reduce leptin concentration over six months in obese adolescents. Haapala et al. [68] also noted that moderate to high-intensity training, combined with reduced sedentary time, led to lower levels of inflammatory biomarkers among children.

The significant improvements in physical performance parameters—such as the 6MWD, the TUG test, and the symmetry index—along with the inverse correlation between the symmetry index and serum leptin levels highlight the effectiveness of the kinesiotherapy program in managing overweight and obese children.

Asymmetrical loading of the joints leads to a changed distribution of forces in the lower limb joints [69]. Our findings underscore the effectiveness of the kinesiotherapy program in enhancing physical performance, regardless of age, gender, or location. The significant improvements in the TUG test and symmetry index in the SG group highlight the importance of coordination exercises in the program. A higher walking cadence among children in the SG group helps reduce biomechanical strain on the lower limbs and improves coordination, which is particularly beneficial for overweight children in supporting their overall development.

Research on how physical exercise affects serum leptin levels in children with obesity is relatively scarce compared to similar studies in adults, despite the fact that changes in leptin expression are associated with severe obesity [70]. Most studies show that engaging in a structured exercise program leads to reduced leptin levels in conjunction with weight and fat mass control [31,71]. Our research confirms a reduction in serum leptin levels, regardless of changes in BMI, even though none of the children experienced weight gain.

There are clear issues related to differences among various categories of obese children. Research exists on potential factors contributing to obesity based on environment [72,73,74], gender [75], and socio-cultural level [76,77]. However, no studies in the scientific literature that assess different clinical–functional aspects and laboratory markers of inflammatory status in overweight and obese children after a physical therapy program were found, based on the factors mentioned above. We believe that the results of this study, which show the effectiveness of physical therapy, particularly in children aged 6 to 10, especially in girls and those from urban areas, bring a new perspective to this field of research. The present research stands out because it includes inflammatory markers as indicators for future obesity and other metabolic or rheumatological diseases in adulthood. It also evaluates physical activity in two ways: through participants’ self-reports and by using objective assessments of the functional status of the study group. Undoubtedly, this serves as a starting point for further exploration of this growing global issue.

Like recent findings in adults [78,79,80], the authors of this study advocate for combining various types of kinesiotherapy in overweight children to improve the parameters we analyzed. Involving specialists from different fields as part of a multidisciplinary medical team allowed for a more effective and rapid approach to managing overweight children. Overall, the critical role of the immune system in the development of obesity-related inflammation, as well as the effectiveness of kinesiotherapy as a non-pharmacological anti-inflammatory intervention, should not be overlooked [63].

Overall, physical performance improved in the study group, showing statistically significant gains. However, certain results—like walking cadence and symmetry index for rural children and symmetry index for older children—did not reach statistical significance. Incorporating coordination and flexibility exercises is essential for enhancing and maintaining a healthy gait pattern in overweight or obese children. The lack of significant differences in 6MWD and TUG values between the two assessment points for both groups suggests that these measures are influenced by multiple factors beyond a supervised exercise program, unlike parameters such as walking cadence and symmetry index.

When comparing age, sex, and environment between groups in the second evaluation, few differences emerged—primarily in 6MWD for older school-aged children and walking cadence across age groups. This finding points to the complexity of physical performance in children, which is shaped by individual growth and developmental factors, regardless of their environment. Despite these findings, the current study has some *limitations*.

The literature emphasizes the importance of evaluating blood markers associated with a reduced inflammatory state (such as the neutrophil-to-lymphocyte ratio, CRP, fibrinogen, and various cytokines and adipokines) for the early detection of inflammation related to nutritional disorders. Indeed, identifying other important markers like IL-6, TNF-alpha, or adiponectin gives a clearer understanding of the inflammatory status. The research was based on the idea that CRP and leptin are commonly used as screening tests in patient evaluations in hospitals around the world. Furthermore, leptin and CRP are often measured together to give a more complete view of inflammation in obese individuals. In contrast, IL-6 and TNF-alpha are more specifically related to immune cell activation, while adiponectin serves as an anti-inflammatory marker. Our study may lack the statistical power needed to detect meaningful differences in leptin levels, but the results obtained provide an indication of the specified effects. Changes in BMI were also not tracked, as the 3-month monitoring period may not have been long enough to yield significant and sustained results. Moreover, we did not quantify eating habits and lifestyle factors, which could have influenced the results and added more depth to the correlations made. Certainly, long-term follow-up is much more realistic for assessing the impact of lifestyle interventions on inflammation and physical fitness. It is one of the future objectives of our team. In our country, collaboration is being perfected between medical staff in the educational and healthcare systems to ensure comprehensive monitoring (clinical, paraclinical, and functional) throughout the entire schooling period of overweight and obese children.

Another limitation was the lack of research on the effectiveness of physical therapy, assessed through clinical and biological parameters, in groups of overweight or obese children based on age, gender, and environment of origin, which made comparisons in Section 4 challenging.

Regarding the internal validity of this study, the authors believe it was strengthened by accounting for factors such as anthropometric measurements, biological markers of inflammation, and functional parameters like gait analysis, all evaluated using objective tests and measurements. The internal bias was further minimized by having comparable patient group sizes, including only children, and using the same team of specialists for all evaluations.

As for external validity, the results can be generalized, as a significant portion of the global child population shares the same characteristics that contribute to obesity and being overweight (poor diet and lack of physical activity).

One limitation of our study is also the absence of a multivariate analysis to dissect the complex relationships among inflammatory markers, physical fitness measures, and demographic characteristics such as gender, age, and environment. The relatively small sample size and the study design constrained our ability to perform such analyses without compromising statistical power. Future research with larger cohorts and comprehensive data collection is necessary to employ multivariate methods that can control for confounding variables and provide a more precise interpretation of these relationships.

While our findings indicate improvements in physical performance and reductions in inflammatory markers following the kinesiotherapy program, we recognize that the interplay between these outcomes and demographic factors is multifaceted. The trends observed suggest that age, gender, and environment may influence the effectiveness of interventions, but without multivariate analysis, we cannot definitively attribute these effects to specific variables. This highlights the need for future studies to utilize multivariate approaches to better understand and interpret these complex relationships.

## 5. Conclusions

In conclusion, this study demonstrates that a structured kinesiotherapy program significantly enhances physical performance and reduces inflammation in overweight and obese children. Regular aerobic and coordination exercises led to decreases in C-reactive protein (CRP) and serum leptin levels, key markers of subclinical inflammation. Benefits varied by age, gender, and environment, with children aged 6 to 10, especially girls and those from urban areas, showing the most improvement. This highlights the importance of early, tailored interventions in managing childhood obesity effectively. Key actionable insights include early intervention (start exercise programs early to maximize benefits), personalized programs (design age-, gender-, and environment-specific exercise routines), comprehensive exercise (combine aerobic, strength, coordination, and flexibility exercises for optimal results), regular monitoring (track CRP and leptin levels to adjust programs as needed), and multidisciplinary approach (collaborate with various specialists to support sustainable lifestyle changes). By linking inflammation markers with physical fitness and considering demographic factors, this study provides valuable guidance for developing effective obesity interventions in children. Future research should explore these relationships over longer periods and include additional lifestyle factors to further refine intervention strategies.

## Figures and Tables

**Table 2:** Anthropometric data at the T1 moment.

	SG 75 Children		CG 65 Children		*p*-Value
Age (years)	9.22 (3.5)		9.89 (2.3)		0.1347
BMI (kg/m^2^)	29.22 (4.1) (25.6–38.4)		29.32 (3.5) (25.8–35.5)		0.2859
	**n (%)**	**BMI (kg/m^2^)**	**n (%)**	**BMI (kg/m^2^)**	
Girls	33 (44%)	29.06 (4.3)	33 (51%)	29.67 (3.4)	0.7707
Boys	42 (56%)	29.34 (4.0)	32 (49%)	28.96 (3.6)	0.4158
Urban	36 (48%)	29.75 (4.4)	34 (54%)	29.45 (3.4)	0.2876
Rural	39 (52%)	28.73 (3.8)	31 (46%)	29.18 (3.7)	0.7612
Younger school children	53 (65.33%)	29.95 (4.3)	37(47.70%)	28.79 (3.2)	0.3579
Older school children	22 (34.66%)	29.86 (3.6)	28 (52.30%)	30.03 (3.8)	0.1700

Variables are reported as mean (SD) (standard deviation), n = number of subjects. Younger schoolchildren = 6–10 years, older schoolchildren = 11–15 years, BMI = body mass index. *p*= the t-test for independent samples.

**Table 3:** Laboratory test results.

Laboratory Tests		Mean	SD	*p*-Value
Glycemia T1 (mg/dL)	SG	82.48 * ^&^	12.46	* = 0.0474 ^&^ = 0.0509
	CG	78.63 &	10.66	
Glycemia T2 (mg/dL)	SG	81.04 *	9.42	
	CG	79.97	10.39	
Total cholesterol T1 (mg/dL)	SG	145.91 *	17.84	* = 0.0065 ^#^ = 0.043 ^&^ = 0.0091
	CG	145.42 ^#^	13.86	
Total cholesterol T2 (mg/dL)	SG	141.85 * ^&^	12.96	
	CG	151.74 ^# &^	27.35	
Triglycerides T1 (mg/dL)	SG	94.40 *	35.76	* = 0.000
	CG	95.18	28.33	
Triglycerides T2 (mg/dL)	SG	87.89 *	32.19	
	CG	95.92	26.98	

SG = study group, CG = control group; SD = standard deviation, * = *p* value when comparing initial—final SG mean values for the same parameter, paired-sample Student’s *t*-tests, ^#^ = *p* value when comparing initial—final CG mean values for the same parameter, paired-sample Student’s *t*-tests, ^&^ = *p* value when comparing mean values between the two groups in T1 for the same parameter, the t-test for independent samples, The most important significant correlations were marked with bold.

**Table 4:** Leptin and CRP—standard test values.

	Leptin			CRP		
	T1	T2	*p*-Value	T1	T2	*p*-Value
**Study group** (r = 0.5642, correlation between average leptin values, r = 0.986, correlation between average CRP values)						
Mean (SD)	23.02 (7.17) * ^&^	16.62 (7.83) * ^@^	* = 0.000 **^&^ = 0.0164** **^@^ = 0.0009**	4.13 (1.00) * ^&^	3.90 (0.95) * ^@^	* = 0.000 **^&^ = 0.0261** **^@^ = 0.0000**
			
			
Median value	**22.6**	**16.3**		**4.5**	**3.74**	
The Wilcoxon signed-rank test results indicate that there is no significant difference between the mean and median values for leptin and CRP in the SG, as all *p*-values are 1.0. This suggests that the data distribution for these parameters is relatively symmetric, and both mean and median can be used for interpretation.						
Boys (42)	22.48 (6.5) *	15.83 (7.3) * ^@^	* = 0.0000	4.14 (1.0) *	3.93 (1.0) * ^@^	* = 0.000 ^@^ = 0.0022
			^@^ = 0.0019			
Girls (33)	23.78 (8.0) *	17.62 (8.5) *	* = 0.0001	4.14 (1.1) *	3.87 (1.0) * ^@^	* = 0.0000 ^@^ = 0.0001
Urban (36)	23.64 (8.2) *	17.07 (8.5) * ^@^	* = 0.000 ^@^ = 0.03	4.40 (1.1) *	4.13 (1.0) * ^@^	* = 0.000 ^@^ = 0.005
Rural (39)	22.45 (6.1) *	16.20 (7.3) * ^@^	* = 0.000 ^@^ = 0.0072	3.90 (0.9) *	3.70 (0.8) * ^@^	* = 0.000 ^@^ = 0.00004
Younger school children (53) 6–10 years	23.79 (7.3) *	16.91 (7.9) * ^@^	* = 0.000 ^@^ = 0.0020	4.09 (1.0) *	3.88 (1.0) * ^@^	* = 0.000 ^@^ = 0.000
Older school children (22) 11–15 years	21.18 (6.7) *	15.91 (7.8) *	* = 0.01	4.24 (1.0) *	3.96 (0.9) * ^@^	* = 0.000 ^@^ = 0.000
**Control group** (r = 0.716, correlation between average leptin values, r = 0.987, correlation between average CRP values)						
Mean (SD)	**20.39 (5.65) ^&^**	**20.49 (5.69) ^@^**	**^&^ = 0.0164** **^@^ = 0.0009**	**4.50 (0.92) ^&^**	**4.70 (0.89) ^@^**	**^&^ = 0.0261** **^@^ = 0.0000**
Median value	**21.01**	**21.6**		**5.05**	**4.85**	
The Wilcoxon signed-rank test results indicate that there is no significant difference between the mean and median values for leptin and CRP in the CG, as all *p*-values are 1.0. This suggests that the data distribution for these parameters is relatively symmetric, and both mean and median can be used for interpretation.						
Boys (32)	20.33 (5.0) ^#^	20.43 (5.0) ^# @^	^#^ = 0.004 ^@^ = 0.0019	4.43 (1.0) ^#^	4.63 (0.9) ^# @^	^#^ = 0.000 ^@^ = 0.0022
Girls (33)	20.45 (6.3)	20.56 (6.4)	0.1209	4.58 (0.9) ^#^	4.77 (0.9) ^# @^	^#^ = 0.0000 ^@^ = 0.0001
Urban (34)	20.12 (4.8) ^#^	20.24 (4.9) ^# @^	^#^ = 0.001 ^@^ = 0.03	4.6 (0.9) ^#^	4.79 (0.9) ^# @^	^#^ = 0.000 ^@^ = 0.005
Rural (31)	20.68 (6.5)	20.77 (6.5) ^@^	^@^ = 0.0072	4.40 (0.9) ^#^	4.59 (0.9) ^# @^	^#^ = 0.000 ^@^ = 0.00004
Younger school children (53) 6–10 years	21.19 (5.4)	21.36 (5.4) ^@^	^@^ = 0.0020	4.47 (0.8)	4.67 (0.8) ^@^	^@^ = 0.000
Older school children (22) 11–15 years	19.33 (6.0)	19.36 (6.0)	0.0945	4.55 (1.0)	4.74 (1.0) ^@^	^@^ = 0.000
					

SD = Standard deviation; CRP = C-reactive protein; * = *p* value when comparing average initial—final SG values for the same parameter, paired-sample Student’s *t*-tests; ^#^ = *p* value when comparing initial—final CG average values for the same parameter, paired-sample Student’s *t*-tests; ^&^ = *p* value when comparing the average values between the two groups at the moment T1 for the same parameter, the *t*-test for independent samples; ^@^ = *p* value when comparing the average values between the two groups at the moment T2 for the same parameter, the *t*-test for independent samples.

**Table 5:** Six minutes walking test results for both groups.

	Six Minutes Walking Test					
	6MWD (Distance Walked in 6 min—m)			Walking Cadence (Steps/min)		
	T1	T2	*p*-Value	T1	T2	*p*-Value
**Study group (75 children)**						
Mean (SD)	401.73 (70.83) *	422.64 (82.86) *	* = 0.000	119.56 (12.43) *	122.97 (12.67) * ^@^	* = 0.000 **^@^ = 0.0410**
				
Median value	**394**	**408**		**122**	**126**	
The Wilcoxon signed-rank test results indicate that there is no significant difference between the mean and median values for 6 MWD and cadence in the SG group, as all *p*-values are 1.0. This suggests that the data distribution for these parameters is relatively symmetric, and both mean and median can be used for interpretation.						
Boys (n (%) = 42 (56%))	414.39 (71.54) *	434.87 (72.25) *	* = 0.000	117.91 (13.7) *	121.17 (13.8) *	* = 0.000
Girls (n (%) = 33 (44%))	380.28 (67.45) *	408.60 (79.18) *	* = 0.000	121.67 (10.3) *	125.26 (10.8) * ^@^	* = 0.000 ^@^ = 0.018
Urban (n (%) = 36 (48%))	392.23 (73.04) *	412.55 (74.64) *	* = 0.000	120.19 (12.2) *	123.58 (12.8) *	* = 0.000
Rural (n (%) = 39 (52%))	402.91 (85.81) *	436.71 (88.41) *	* = 0.000	118.99 (12.7)	122.41 (12.7)	0.6322
Younger school children (n (%) = 53 (65.33%))	379.62 (75.52) *	408.19 (77.53) *	* = 0.0000	124.03 (8.9) ^&^	127.6 (9.2) ^@^	^&^= 0.0000 ^@^ = 0.005
Older school children (n (%) = 22 (34.66%))	419.72 (87.81) *	441.56 (87.82) * ^@^	* = 0.0000 ^@^ = 0.000	108.80 (13.3) *	111.82 (13.1) * ^@^	* = 0.0000 ^@^ = 0.032
**Control group (65 children)**						
Mean (SD)	**407.73 (58.45) ^#^**	**411.92 (59.15) ^#^**	**^#^ = 0.000**	**117.51 (11.85) ^#^**	**118.70 (11.81) ^@^**	**^#^ = 0.143** **^@^ = 0.0410**
Median value	**388.5**	**395**		**119**	**120.5**	
The Wilcoxon signed-rank test results indicate that there is no significant difference between the mean and median values for 6 MWD and cadence in the CG group, as all *p*-values are 1.0. This suggests that the data distribution for these parameters is relatively symmetric, and both mean and median can be used for interpretation.						
Boys (n (%) = 32 (49%))	417.15 (79.47) ^#^	419.89 (91.20) ^#^	^#^ = 0.000	117.75 (11.7)	118.98 (12.6)	0.0826
Girls (n (%) = 33 (51%))	383.86 (89.45) ^#^	387.52 (79.27) ^#^	^#^ = 0.000	117.27 (12.1) ^#^	118.42 (12.2) ^# @^	^#^ = 0.000 ^@^ = 0.018
Urban (n (%) = 34 (54%))	394.59 (70.46) ^#^	398.90 (70.34) ^#^	^#^ = 0.000	117.53 (11.3) ^#^	118.75 (11.2) ^#^	^#^ = 0.000
Rural (n (%) = 31 (46%))	416.45 (88.47) ^#^	419.55 (85.44) ^#^	^#^ = 0.000	117.48 (12.4)	118.65 (12.6)	0.5133
Younger school children (n (%) = 37 (47.70%))	383.00 (79.84) ^#^	386.24 (70.13) ^#^	^#^ = 0.000	117.95 (11.7) ^&^	119.32 (11.6) ^@^	^&^ = 0.009 ^@^ = 0.005
Older school children (n (%) = 28 (52.30%))	418.75 (88.15) ^#^	421.92 (77.25) ^# @^	^#^ = 0.000 ^@^ = 0.000	116.93 (12.3)	117.88 (12.2) ^@^	^@^ = 0.032

SD = standard deviation, n = no. of subjects, * = *p* value when comparing initial—final SG mean values for the same parameter, paired-sample Student’s *t*-tests, ^#^ = *p* value when comparing initial—final CG mean values for the same parameter, paired-sample Student’s *t*-tests, ^&^ = *p* value when comparing mean values between the two groups in T1 for the same parameter, the *t*-test for independent samples, ^@^ = *p* value when comparing mean values between the two groups in T2 for the same parameter, the t-test for independent samples.

**Table 6:** Symmetry index and Time Up and Go test results for both groups.

	Time Up and Go (s)			Symmetry Index (Percent)		
	T1	T2	*p*-Value	T1	T2	*p*-Value
**Study group (75 children)**						
Mean (SD)	15.64 (2.26) *	13.93 (2.16) *	* = 0.000	**91.67 (4.93)**	**91.88 (10.84) ^@^**	**^@^ = 0.0240**
Median value	**15.3**	**13.6**		**93.1**	**94.8**	
The Wilcoxon signed-rank test results indicate that there is no significant difference between the mean and median values for TUG and Symmetry index in the SG group, as all *p*-values are 1.0. This suggests that the data distribution for these parameters is relatively symmetric, and both mean and median can be used for interpretation.						
Boys (n (%) = 42 (56%))	15.46 (1.7) *	13.73 (1.6) *	* = 0.000	92.68(4.7) *	93.88 (4.2) * ^@^	^@^ = 0.0471
Girls (n (%) = 33 (44%))	15.87 (2.8) *	14.20 (2.7) *	* = 0.000	90.40 (5.0) ^&^	89.36 (15.4)	^&^ = 0.0225
Urban (n (%) = 36 (48%))	15.23 (1.5) *	13.56 (1.5) *	* = 0.000	90.97(5.6) *	92.39 (4.9) *	* = 0.000
Rural (n (%) = 39 (52%))	16.02(2.7) *	14.28(2.6) *	* = 0.000	92.33 (4.2)	91.40 (14.4)	0.6233
Younger school children (n (%) = 53 (65.33%))	15.96 (2.5) *	14.22 (2.4) *	* = 0.0000	90.84 (5.2) *	92.41 (4.8) *	* = 0.0000
Older school children (n (%) = 22 (34.66%))	14.86 (1.4) *	13.24 (1.2) *	* = 0.0000	93.70 (3.5)	90.6 (18.9)	0.4677
**Control group (65 children)**						
Mean (SD)	**17.37 (12.85)**	**16.42 (12.64)**	**0.074**	**91.96 (3.11)**	**86.55 (20.76) ^@^**	**0.472** **^@^ = 0.0240**
Median value	**16.17**	**15.84**		**92.05**	**91.03**	
The Wilcoxon signed-rank test results indicate that there is no significant difference between the mean and median values for TUG and Symmetry Index in the CG group, as all *p*-values are 1.0. This suggests that the data distribution for these parameters is relatively symmetric, and both mean and median can be used for interpretation.						
Boys (n (%) = 32 (49%))	18.96 (1.6)	18.72 (1.5)	0.0981	91.13 (3.2)	87.77 (16.4) ^@^	^@^ = 0.0471
Girls (n (%) = 33 (51%))	15.83 (4.9) ^#^	14.19 (3.6) ^#^	^#^ = 0.000	92.76 (2.9) ^&^	85.36 (24.5)	^&^ = 0.0225
Urban (n (%) = 34 (54%))	19.08 (1.7)	17.75 (1.7)	0.2135	92.21(3.4)	86.42 (21.1)	0.2598
Rural (n (%) = 31 (46%))	15.50 (1.8)	14.96 (3.1)	0.3421	91.67 (2.8)	86.68 (20.7)	0.4418
Younger school children (n (%) = 37 (47.70%))	16.16 (4.7)	14.95 (2.8)	0.5055	91.37 (3.2)	88.51 (15.4)	0.1430
Older school children (n (%) = 28 (52.30%))	18.24 (11.5)	18.36 (16)	0.2601	92.73 (2.9)	83.95 (26.4)	0.3267
					

SD = standard deviation, n = no. of subjects, * = *p* value when comparing initial—final SG mean values for the same parameter, paired-sample Student’s *t*-tests, ^#^ = *p* value when comparing initial—final CG mean values for the same parameter, paired-sample Student’s *t*-tests, ^&^ = *p* value when comparing mean values between the two groups in T1 for the same parameter, the *t*-test for independent samples, ^@^ = *p* value when comparing mean values between the two groups in T2 for the same parameter, the *t*-test for independent samples.

## Data Availability

Data are contained within the article.
